# Teacher punishment intensity and parental trust in rural China: a moderated mediation of violation severity and trustworthiness

**DOI:** 10.3389/fpsyg.2025.1572656

**Published:** 2025-05-21

**Authors:** Zhen Zhang, Yali Zhao, Xiaoyu Huang, Juan Guo, Chunhui Qi

**Affiliations:** ^1^Faculty of Education, Henan Normal University, Xinxiang, China; ^2^Faculty of Education, Henan University, Kaifeng, China

**Keywords:** punishment intensity, violation severity, parental trust, trustworthiness, rural China

## Abstract

Teacher discipline functions as an essential instrument not only for promoting the healthy development of adolescents but also for cultivating the trust relationship between parents and educators. Based on signaling theory and just deserts theory, this study explore the effects of teacher discipline intensity, student violation severity, and perceived trustworthiness on parental trust in rural China. A total of 1,206 parents residing in rural areas of Yichuan County, Henan Province, China, completed an online questionnaire. The findings indicate that disciplinary intensity exhibit a significant negative correlation with parental trust. Trustworthiness (ability, benevolence, and integrity) completely mediated this negative effect of disciplinary intensity on parental trust. Moreover, violation severity moderated the mediation model. Specifically, teacher discipline intensity significantly negatively predicts parents’ perception of teachers’ ability, benevolence, and integrity under low violation conditions. In contrast, disciplinary intensity can also significantly negatively predict integrity; however, it was not possible to predict ability or benevolence in high violation conditions. These findings provide educators with insights on implementing appropriate discipline to enhance parental trust.

## 1 Introduction

The mutual trust between teachers and parents serves as the fundamental cornerstone that facilitates home-school cooperation and parental involvement in education, permeating throughout the entire school life of children and adolescents (Hummel et al., 2023a,b; Zhang et al., 2024). Trust, acting as a lubricant for home-school collaboration, not only plays a crucial role in initiating, establishing, and maintaining positive home-school relationships but also contributes to the thriving development of students, classes, and schools (Bormann et al., 2021; Niedlich et al., 2021; Shayo et al., 2021). Over the past three decades, the emergence, development, and influencing factors of home-school trust have emerged as a research hotspot across various disciplines, including education (Niedlich et al., 2021), psychology (Shayo et al., 2021), and sociology (Bormann et al., 2021), and have been extensively, profoundly, and systematically explored (Rautamies et al., 2021; Santiago et al., 2016; Uitto et al., 2021). As an integral component of home-school trust, parents’ trust in teachers (i.e., parental trust) refers to the willingness and behavior of parents to voluntarily entrust their children’s education to teachers based on confidence in teachers’ ability, benevolence, competence (Hiatt et al., 2023). The essence of parental trust fundamentally involves acknowledging and accepting the vulnerability inherent in teachers. This willingness or action to embrace the vulnerability of others and take risks within interpersonal relationships is referred to as behavioral trust (Gillespie, 2015). Trust manifests in various forms and can be assessed through multiple methodologies, including psychological scales, risk-taking behaviors, sharing of secrets, behavioral game tasks, and suggestion adoption, among others (Legood et al., 2023). Extensive studies using qualitative methods, surveys, and other quantitative techniques have consistently found that most parents place significant trust in their children’s teachers (Huang, 2022; Janssen et al., 2012; Schuster et al., 2025). Furthermore, parents’ trust is influenced by individual, family, teacher-related, and school-related factors (Adams and Christenson, 2000; Bower et al., 2011; Forsyth et al., 2006; Kikas et al., 2011, 2016; Lerkkanen and Pakarinen, 2021).

During the interaction between parents and teachers, parents frequently evaluate teachers’ trustworthiness based on various social cues, such as gender characteristics, professional competence, and classroom management styles (Kikas et al., 2011, 2016; Schuster et al., 2025). Among these factors, teacher discipline has consistently been a focal point of concern for families, schools, and society, significantly influencing parents’ trust in teachers. In recent years, behavioral economics has seen a surge in research based on economic game theory. Through rigorous experimental design and data analysis, studies have demonstrated that third-party punishment substantially affects observers’ trust evaluations of punishers and their subsequent behaviors (Jordan et al., 2016; Salcedo and Jimenez-Leal, 2024; Sun et al., 2023). In the context of enterprise management, Wang and Murnighan (2017) found that moderate disciplinary actions by supervisors can enhance observers’ trust in supervisors and influence their decision-making. This finding has also been corroborated by recent studies (Li et al., 2024; Spadaro et al., 2023). More importantly, Zhang and Qi (2024) observed similar effects in school management contexts. Despite the substantial achievements of existing research, the specific pathways and mechanisms by which teacher discipline influences parental trust within home-school interactions remain unclear. Given that parental trust is crucial for fostering deep collaboration between home and school, there is an urgent need for systematic research to precisely analyze how teacher discipline predicts parental trust in educational settings, thereby providing a solid theoretical foundation and practical guidance for optimizing home-school relationships.

## 2 Literature review and research hypotheses

### 2.1 Disciplinary intensity and parental trust

Educational discipline refers to the means by which teachers, in accordance with educational regulations, manage and instruct students who have violated discipline, facilitating their recognition and rectification of mistakes (Zhang and Qi, 2024). In the school management context, rewards and punishments are the most frequently employed measures by teachers for student management. In the legal regulations of countries worldwide, the scope of disciplinary intensity is rather extensive, ranging from mild verbal education to the most severe suspension or expulsion from school. When implementing discipline, it not only directly influences the cognition and behavior of the students who have violated the rules but also has an indirect effect on observing students and even parents (Sun et al., 2023; Wang and Murnighan, 2017; Zhang and Qi, 2024). Although the degree of disciplinary intensity varies significantly, all disciplinary actions will have an impact on the social evaluation of the punisher, the punished, or the bystanders. According to the core perspective of the signaling theory, every word and action of the disciplinarian conveys their moral character to the punished and the bystanders, thereby influencing the trustworthiness judgment of the disciplinarian by the punished and the bystanders (Connelly et al., 2011; Dhaliwal et al., 2022; Gintis et al., 2001). In the daily management of schools, discipline is an important means to guide students to adhere to social norms, emphasizing the prevention and correction of deviant behaviors rather than retaliating against students’ misbehaviors. Based on this point of departure, excessive and frequent discipline might imply callousness, thereby weakening the interpersonal trust that bystanders have in the disciplinarian (Engeler and Raihani, 2024; Horita, 2010; Kiyonari and Barclay, 2008; Raihani and Bshary, 2019; Spadaro et al., 2023). Some studies have discovered that people often consider punishers to be disagreeable and untrustworthy, and excessive or selfish punishment will reduce the trust of bystanders (Sun et al., 2023; Wang and Murnighan, 2017; Zhang and Qi, 2024). In light of this, we formulate research hypothesis 1: Disciplinary intensity can negatively predict parental trust.

### 2.2 Trustworthiness as a mediator

Trustworthiness refers to an individual’s consistent ability to fulfill the expectations of others in specific behaviors, serving as a proximal antecedent variable of trust (Zhang et al., 2024). Unlike trust, trustworthiness reflects a positive expectation that another party will perform a particular action. Gillespie (2015) elaborates on the distinction between trust and trustworthiness by arguing that, although an individual may perceive another as trustworthy, this perception does not necessarily translate into actual trusting behavior toward that person. Structurally, trustworthiness comprises three fundamental dimensions: ability, benevolence, and integrity (Hiatt et al., 2023; Mayer and Davis, 1999). Extensive research has consistently demonstrated that individuals tend to evaluate or predict interpersonal trust in leaders, teachers, or managers based on these dimensions (Colquitt et al., 2007; Hiatt et al., 2023; Zhang and Qi, 2024). In accordance with signaling theory, the punisher’s actions can effectively communicate their moral character and concerns for reputation to observers (Jordan et al., 2016; Jordan, 2023). Excessive discipline is frequently attributed by observers to negative attributes such as insensitivity, malevolence, and a deficiency in empathy. Several empirical studies have demonstrated that perceived trustworthiness serves as a mediator in the relationship between punitive behavior and bystander trust (Sun et al., 2023; Wang and Murnighan, 2017; Zhang and Qi, 2024; Zhang et al., 2025a). For instance, Wang and Murnighan (2017) demonstrated that stringent disciplinary actions imposed by mangers can diminish bystanders’ interpersonal trust by eroding their perception of trustworthiness. Therefore, we propose hypothesis 2: Trustworthiness may serve as a mediator in the relationship between disciplinary intensity and parental trust.

### 2.3 Violation severity as a moderator

Violation severity pertains to the assessment of the gravity of violations based on factors such as intention, frequency, and consequences of the misconduct (Eriksson et al., 2017; Peterson, 2024). The principle of proportionality in punishment is a fundamental guideline adhered to in administrative penalty practices and holds significant reference value for educators when implementing disciplinary actions. Educators must invariably consider the gravity of a student’s offense when administering punishment. Specifically, minor infractions should be met with proportionate minor penalties, whereas serious violations necessitate more stringent punishments. The just deserts theory (JDT) underscores that punishment should be commensurate with the severity of the violation to ensure its legitimacy. Some empirical studies have found that appropriate punishment can enhance bystander’s perceived trustworthiness, while disproportionate punishment can weaken perceived trustworthiness (Wang and Murnighan, 2017; Peterson, 2024). For instance, one study investigated the impact of disciplinary intensity and violation severity on bystanders’ perceived trustworthiness in a school management context, revealing that suitable discipline enhances bystanders’ trust in teachers, while inappropriate discipline undermines this trust (Zhang and Qi, 2024). Based on these findings, we propose Hypothesis 3: The severity of the violation moderates the negative relationship between disciplinary intensity and perceived trustworthiness.

**Figure 1:**
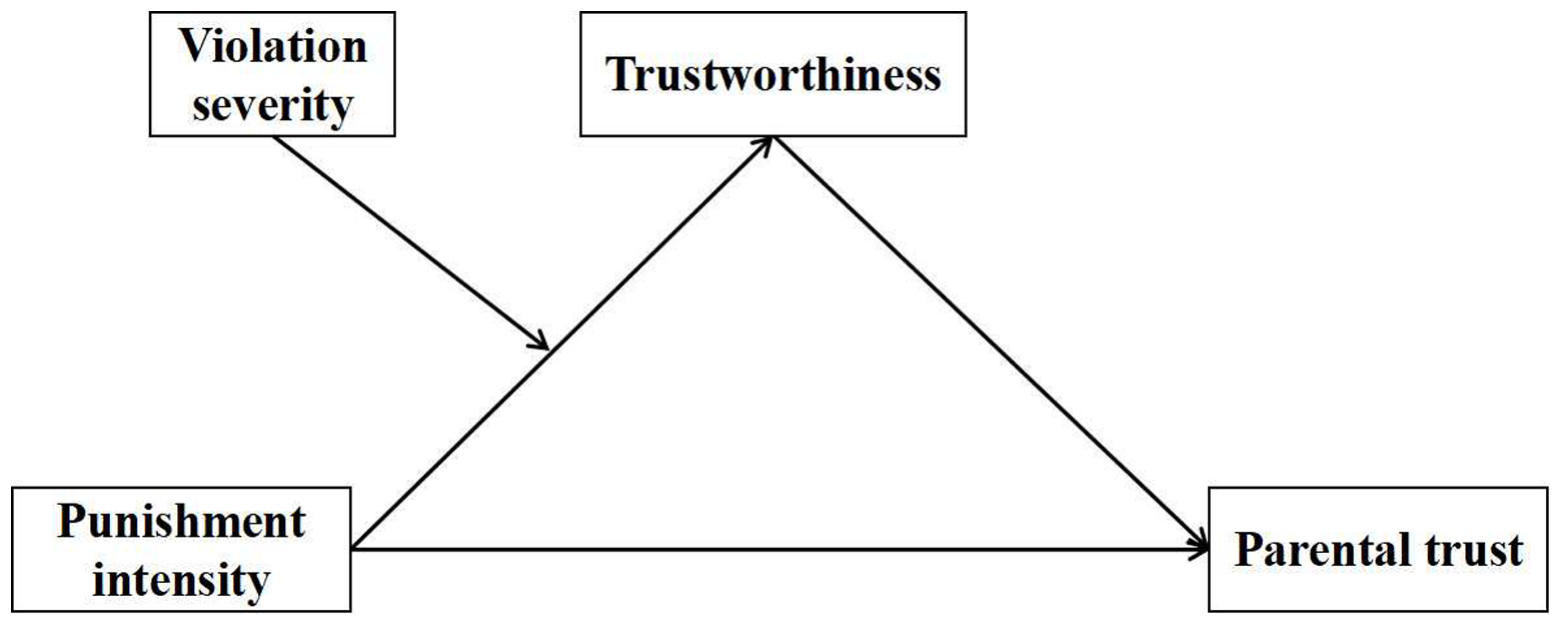
Research model.

To sum up, this study constructed a moderated mediation effect model to comprehensively examine the effects of teacher discipline intensity, student violation severity, and trustworthiness on parents’ trust. The research model is shown in Figure 1.

## 3 Method

### 3.1 Participants and procedure

Consistent with previous studies (Qian et al., 2024; Zhao et al., 2024), in order to obtain the target subjects quickly and efficiently, the convenient sampling method was employed to select a junior middle school in Yichuan County, Luoyang City, Henan Province. Online electronic questionnaires were then distributed to the parents of students in grades 7 and 8 using the same sampling approach. The specific items are shown in Table 1, and the rationale regarding the questions on the questionnaire is discussed in the subsections of 2.2. Similar to previous studies (Qian et al., 2024; Zhao et al., 2024), given the pervasive use of smartphones, the present research utilized an online platform (Questionnaire Star, www.wjx.cn) for data collection to efficiently and expeditiously accomplish the testing tasks. Specifically, the head teacher shared the questionnaire link in the class parent communication group and requested that students remind their parents to complete the survey over the weekend. In total, 1,230 questionnaires were collected, of which 1,206 were valid, yielding an effective response rate of 98.05%. Among the valid samples, 38% of the respondents were male parents with an average age of 40.63 years. Among their children, 52% were male, 81% were in the 7th grade, and 94% were non-only children. The average age of the children was 12.57 years, with approximately 4% being boarding students. Most households exhibited residential stability and favorable economic conditions (see Table 1). The study was approved by the Ethics Committee of the Faculty of Education, Henan Normal University.

**Table 1:** The explanatory and descriptive statistics of variables in data analysis.

Type	Variable name	Items	Variable description	*M*	SD
Predictor variable	Punishment intensity	Class teacher often criticizes me	1 = Strongly disagree; 2 = disagree; 3 = Agree; 4 = Strongly agree	1.26	0.54
		My parents often receive criticism about me from the teacher			
Outcome variable	Parental trust	Do you think the teacher is responsible for your child?	1 = Not at all; 2 = Not very; 3 = Average; 4 = Quite; 5 = Very	4.56	0.82
		Do you think the teacher is patient with your child?			
Mediation variable	Ability	The class teacher is fully capable of performing his or her duties effectively	1 = Not at all; 2 = Not very; 3 = Average; 4 = Quite; 5 = Very	4.52	0.78
		I have confidence in the class teacher’s professional capabilities			
	Benevolence	The class teacher demonstrates significant concern for your child		4.42	0.85
		The class teacher will make every effort to assist your child			
	Integrity	The class teacher is a fair individual		4.49	0.80
		The class teacher’s actions are consistent with reasonable standards			
Moderator variable	Violation severity	I often arrive late	1 = Strongly disagree; 2 = disagree; 3 = Agree; 4 = Strongly agree	1.08	0.34
		I often skip classes			
Control variable	Students characteristics	Student gender	0 = Female; 1 = Male	1.48	0.50
		Student age	Age at the time of the survey	12.57	0.65
		Student grade	0 = grade 7; 1 = grade 9	1.19	0.40
		Boarding status	0 = Not boarding; 1 = boarding	1.95	0.23
		Only child status	1 = Only child; 2 = Not an only child	1.96	0.20
	Parental characteristics	Parental identity	1 = Biological father; 2 = Biological mother	1.62	0.49
		Parental age	Age at the time of the survey	40.63	5.08
		Parental education level	1 = primary education and below; 2 = lower secondary education; 3 = high school education; 4 = college education; 5 = Graduate education	3.12	0.89
	Family characteristics	Family migration status	1 = Non-migrated; 2 = Intra-provincial migration; 3 = Inter-provincial migration	0.17	0.26
		Family economic status	1 = Difficult; 2 = Medium; 3 = Rich	1.81	0.39

### 3.2 Measures

#### 3.2.1 Disciplinary intensity

Drawing upon existing literature (Zhang and Qi, 2024; Lewis et al., 2005), the present study utilizes two specifically designed questions to systematically assess the intensity of teacher discipline: “The head teacher often criticizes your child” and “You often receive criticism from the other teacher.” Participants rate their responses to these statements using a four-point Likert scale, where options extend from 1 (“strongly disagree”) to 4 (“strongly agree”). The average score of the two items is used as the score for disciplinary intensity. An increased score reflects a higher perception of disciplinary intensity. The reliability of this measurement, as assessed by Cronbach’s alpha, is reported at 0.66.

#### 3.2.2 Parental trust

Building on prior research (Zhang et al., 2025b; Lerkkanen and Pakarinen, 2021), this study employs two purposefully crafted items to comprehensively evaluate parental trust: “Do you feel that the teacher is responsible for your child?” and “Do you think the teacher will be patient with your child?” Respondents provided their ratings on a five-point Likert scale, ranging from 1 (representing “not responsible at all” or “not patient at all”) to 5 (representing “very responsible” or “very patient”). The average score of the two items is used as the score for parental trust. Higher scores suggested increased levels of parental trust in teachers. The consistency of the scale, evaluated using Cronbach’s alpha, was determined to be 0.95.

#### 3.2.3 Trustworthiness

Based on previous research (Jones and Shah, 2016; Zhang and Qi, 2024) and the background of Chinese culture, two most relevant items from each sub-dimension of the trustworthiness scale developed by Hiatt et al. (2023) were selected to evaluate parents’ perceptions of the class teacher’s ability, benevolence, and integrity. Specifically, to assess parents’ perception of the class teacher’s ability, the following items were used: “The class teacher is fully capable of performing his or her duties effectively” and “I have confidence in the class teacher’s professional capabilities.” To evaluate parents’ perception of the class teacher’s benevolence, the items included: “The class teacher demonstrates significant concern for your child” and “The class teacher will make every effort to assist your child.” For gauging parents’ perception of the class teacher’s integrity, the items were: “The class teacher is a fair individual” and “The class teacher’s actions are consistent with reasonable standards.” Participants rate their responses to these statements using a four-point Likert scale, where options extend from 1 (“strongly disagree”) to 4 (“strongly agree”). The average score of the items is used as the score for each dimension variable. Higher scores indicate greater perceived trustworthiness. The internal consistency coefficients for the ability, benevolence, integrity, and overall trustworthiness scales were 0.95, 0.96, 0.97, and 0.97, respectively.

#### 3.2.4 Violation severity

Building upon earlier research (Zhang and Qi, 2024; Wang and Murnighan, 2017), this study employs two carefully formulated items to comprehensively evaluate the extent of violations: “Your child is often late for class” and “Your child is often absent from class,” Each item rated on a four-point scale. The average score of the two items is used as the score for violation severity. Higher scores indicate a greater frequency of violations, with an internal consistency coefficient of 0.81.

#### 3.2.5 Control variables

Informed by previous studies (Kikas et al., 2011, 2016; Lerkkanen and Pakarinen, 2021), parental trust is influenced by multiple demographic and socioeconomic factors. Therefore, this investigation incorporates a broad spectrum of control variables to address these influences. The selected variables cover student-related factors like gender, age, academic grade, residential status, and whether the child is an only child, as well as parent-related factors such as gender, age, migration status, economic condition, and level of education (see Table 1). To minimize the impact of parental status, the analysis focuses exclusively on data related to biological parents.

### 3.3 Data analysis

For data processing, we employed SPSS version 24.0 along with the PROCESS macro. The analytical procedures are detailed below: First, we conducted common method variance and multicollinearity analysis. Second, we conducted descriptive statistics and correlation assessments for all variables. Following this, the mediation model (Model 4) from the PROCESS macro was applied to investigate the parallel mediation effect by generating the bootstrap 95% confidence interval through 5,000 resampling iterations. Ultimately, to explore the moderated mediation effect, the moderated mediation model (Model 7) of the PROCESS macro was adopted, extracting the bootstrap 95% confidence interval from 5,000 resamples.

## 4 Results

### 4.1 Common method variance and multicollinearity analysis

Factor analysis was conducted on all the items of the measured scale through the Harman single-factor test. It was found that the eigenvalues of the 7 factors were greater than 1, but the interpretation rate of the first common factor was 31.33%, which was lower than 40%. Therefore, the common method bias did not exist. Morevoer, the variance inflation factor (VIF) for all predictors was below 1.33, and the tolerance levels for all predictors were above 0.75, indicating the absence of significant multicollinearity issues.

### 4.2 Descriptive statistics and correlation analysis

A comprehensive statistical analysis was performed to examine the relationships among disciplinary intensity, violation severity, trustworthiness (ability, benevolence, and integrity), and parental trust (see Table 2). The findings indicate a substantial positive relationship between disciplinary intensity and violation severity. Additionally, there is a small negative association between disciplinary intensity and trustworthiness (ability, benevolence, and integrity) and the level of trust from parents. Furthermore, a significant positive correlation was identified between trustworthiness and parental trust. These results indicated a negative relationship between disciplinary intensity and parental trust, which aligns with Hypothesis 1.

**Table 2:** Bivariate correlations matrix of all variables (*N* = 1,206).

Variable	1	2	3	4	5	6
1. Disciplinary intensity	1.00					
2. Violation severity	0.49**	1.00				
3. Ability	−0.08**	−0.03	1.00			
4. Benevolence	−0.08**	−0.03	0.88**	1.00		
5. Integrity	−0.11**	−0.04	0.88**	0.87**	1.00	
6. Parental trust	−0.08**	−0.04	0.85**	0.82**	0.82**	1.00

**p < 0.01.

### 4.3 Mediation effect analysis

To examine the mediating effect of trustworthiness between disciplinary intensity and parental trust, the mediation model (Model 4) was employed for mediation analysis. To minimize potential confounding influences, all demographic variables were incorporated as control factors. These results shown that disciplinary intensity could significantly negatively predict ability (*β* = −0.08, *p* < 0.01), benevolence (*β* = −0.08, *p* < 0.01), and integrity (*β* = −0.11, *p* < 0.01), which in turn was positively related to parental trust (*β* = 0.46, 0.22, and 0.24, *p*s < 0.01). The direct path between disciplinary intensity and parental trust was not significantly. Thus, disciplinary intensity and parental trust are linked completely through ability [*b* = −0.04, 95% *CI* = (−0.08–0.01)], benevolence [*b* = −0.02, 95% *CI* = (−0.04–0.01)], and integrity [*b* = −0.03, 95% *CI* = (−0.06–0.01)]. The mediation effects of ability, benevolence, and integrity were responsible for 45.12, 20.73, and 32.93% of the influence, respectively. Thus, Hypothesis 2 was supported (see Table 3).

**Table 3:** Testing the mediation effect of trustworthiness (ability, benevolence, and integrity) (*N* = 1,206).

Model	Regression equation		Fitting index		Regression coefficient		
	Outcome variable	Predictor variable	*R* ^2^	*F*	*β*	*SE*	*t*
Model 1	Parental trust	Disciplinary intensity	0.02	2.76**	−0.08	0.03	−2.76**
Model 2	Ability	Disciplinary intensity	0.02	2.50**	−0.08	0.03	−2.71**
	Benevolence		0.03	2.81**	−0.08	0.03	−2.74**
	Integrity		0.03	3.14**	−0.11	0.03	−3.89**
Model 3	Parental trust	Disciplinary intensity	0.76	274.30**	−0.00	0.01	−0.05
		Ability			0.46	0.03	13.47**
		Benevolence			0.22	0.03	6.50**
		Integrity			0.24	0.03	7.13**

All variables in the model are brought back into the equation after standardization, **p < 0.01.

### 4.4 Moderated mediation effect analysis

**Figure 2:**
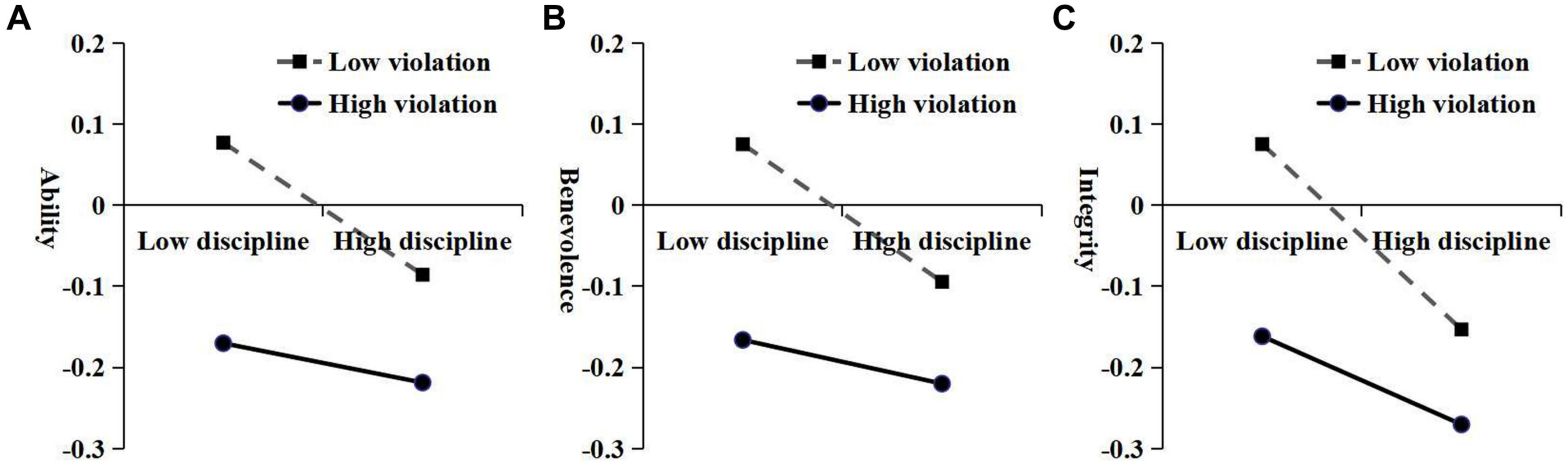
The moderating role of violation severity in the relation between punishment intensity and perceived ability **(A)**, benevolence **(B)**, and integrity **(C)**.

A moderated mediation analysis was performed using the moderated mediation model (Model 7) of the Process 4.0 macro within SPSS version 26.0. A summary of this results was shown in Table 4. The findings shown that the interaction between disciplinary intensity and violation severity had a significantly positive association with ability, benevolence, and integrity (*β* = 0.07, 0.07, and 0.07, *p*s < 0.01). The slope test indicated that the disciplinary intensity can significantly negatively predict parents’ perception of teachers’ ability (*β* = −0.11, *t* = −3.37, *p* < 0.01), benevolence (*β* = −0.12, *t* = −3.52, *p* < 0.01), and integrity (*β* = −0.16, *t* = −4.70, *p* < 0.01) when the severity of violation is low. In contrast, disciplinary intensity can also significantly negatively predict integrity (*β* = −0.08, *t* = −2.18, *p* < 0.05); however, it was not possible to predict parents’ perception of teachers’ ability (*β* = −0.03, *t* = −0.98, *p* > 0.05), or benevolence (*β* = −0.04, *t* = −1.09, *p* > 0.05) (see Figure 2).

**Table 4:** Testing the moderated mediation model (*N* = 1,206).

Model	Regression equation		Fitting index		Regression coefficient		
	Outcome variable	Predictor variable	*R* ^2^	*F*	*β*	*SE*	*t*
Model 1	Parental trust	Disciplinary intensity	0.02	2.76**	−0.08	0.03	−2.76**
Model 2	Ability	Disciplinary intensity	0.04	3.57**	−0.10	0.03	−2.88**
		Violation severity			−0.18	0.05	−3.29**
		Disciplinary intensity × Violation severity			0.07	0.02	4.30**
	Benevolence	Disciplinary intensity	0.04	3.89**	−0.10	0.03	−3.03**
		Violation severity			−0.17	0.05	−3.19**
		Disciplinary intensity × Violation severity			0.07	0.02	4.37**
	Integrity	Disciplinary intensity	0.05	4.28**	−0.14	0.03	−4.21**
		Violation severity			−0.17	0.05	−3.07**
		Disciplinary intensity × Violation severity			0.07	0.02	4.47**
Model 3	Parental trust	Disciplinary intensity	0.76	274.30**	−0.00	0.01	−0.05
		Ability			0.46	0.03	13.47**
		Benevolence			0.22	0.03	6.50**
		Integrity			0.24	0.03	7.13**

All variables in the model are brought back into the equation after standardization, **p < 0.01.

## 5 Discussion

Empirical evidence from current research indicates that the intensity of teacher discipline is a significant negative predictor of parental trust in teachers. Moreover, perceptions of trustworthiness (ability, benevolence, and integrity) fully mediate the negative relationship between disciplinary intensity and parental trust. Finally, this mediation effect is moderated by the severity of student misconduct, with less severe violations leading to a more pronounced mediation effect compared to more serious infractions.

First, our findings support Hypothesis 1 by showing that the intensity of teacher discipline could significantly negatively predict parental trust. This aligns with signaling theory, which suggests that the level of disciplinary actions can predict how much trust parents place in teachers (Dhaliwal et al., 2022; Gintis et al., 2001). As previous studies have noted (Sun et al., 2023; Zhang and Qi, 2024), this outcome can be understood through the perceived reduction in warmth when disciplinary actions are more frequent or severe. The primary goal of teachers’ disciplinary actions is to guide and prevent misbehavior, not to retaliate or harm students. When punishment becomes excessive, it may lead parents to feel that teachers are less warm and approachable, which can affect their trust. Qualitative research highlights that teacher warmth plays a crucial role in building positive relationships between parents and educators (Huang, 2022; Schuster et al., 2025). Therefore, increased disciplinary severity tends to correlate with lower levels of parental trust. Understanding this spillover effect of teacher punishment on parental trust can help broaden educational management practices and provide valuable insights for enhancing home-school collaboration.

Second, Hypothesis 2 is also confirmed, indicating that the intensity of discipline significantly predicts parental trust via perceived trustworthiness (ability, benevolence, and integrity). Consistent with the findings on parental trust, this result aligns with the principles of signaling theory (Dhaliwal et al., 2022; Gintis et al., 2001; Zhang et al., 2025a). Specifically, it suggests that parents can gauge the perceived trustworthiness of teachers based on the intensity of disciplinary actions, thereby determining their level of trust in teachers. In other words, excessive punishment intensity significantly diminishes bystanders’ perceived trustworthiness of the disciplinarian compared to mild punishment intensity, thereby reducing bystanders’ trust. This mediating effect is consistent with previous findings (Wang and Murnighan, 2017; Zhang and Qi, 2024), confirming that trustworthiness serves as a mediator between punishment and bystander trust in both organizational and school management settings. Therefore, excessive discipline can weaken parents’ trust in disciplining teachers by significantly reducing parents’ perceived trustworthiness (ability, benevolence, and integrity).

Third, in alignment with Hypothesis 3, the seriousness of student misconduct moderates the association between the intensity of disciplinary actions and the perceived trustworthiness of teachers (ability, benevolence, and integrity). More specifically, under conditions of minor infractions, a higher level of disciplinary intensity is significantly associated with a decrease in parents’ perceptions of teachers’ ability, benevolence, and integrity. Conversely, in cases of severe violations, while disciplinary intensity still significantly correlates with diminished perceptions of integrity, it is not significantly associated with perceptions of ability or benevolence. As illustrated in Figure 2, the perceived trustworthiness (ability, benevolence, and integrity) remains positive only when teachers impose mild punishments on students with minor violations; otherwise, they are negative. This result aligns with the Just Deserts Theory, which suggests that the harshness of punishment ought to correspond to the gravity of the offense (Mooijman and Graham, 2018). Failing to adhere to this principle can result in skepticism and mistrust among both observers and those who have committed the violation. A substantial body of research utilizing economic game experiments and management simulations has shown that appropriately proportionate sanctions can bolster observers’ trustworthiness in the fairness of the disciplinarian (Wang and Murnighan, 2017; Zhang and Qi, 2024). When violations are severe, the negative relationship between disciplinary intensity and perceived trustworthiness diminishes, possibly because more serious violations raise the threshold for acceptable disciplinary intensity. One interesting result is that disciplinary intensity significantly negatively predicts integrity, but ability and benevolence cannot be predicted in high-violation conditions. It seems that parents may feel that teachers should not resort to frequent disciplinary actions, even when students repeatedly violate rules. Too many instances of discipline might make parents question the teacher’s approach and could affect their perception of the teacher’s fairness and integrity.

Additionally, for the parents of students who frequently violate rules, the severity of disciplinary actions taken by teachers does not significantly correlate with their trust in educators. This highlights a more profound issue regarding home-school collaboration. As illustrated in Figure 2, the interpersonal trust of parents whose children are frequent offenders toward teachers is notably lower than the average. Regardless of the level of punitive actions implemented by teachers, their perceived trustworthiness towards teachers is very low and there is no significant fluctuation in this perception. Fundamentally, this suggests that such parents lack confidence in both the intent and efficacy of teachers’ disciplinary practices. This perceived lack of trustworthiness may arise from prolonged social conditioning. When children frequently breach school discipline, they are more likely to be categorized as troublemakers (Okonofua and Eberhardt, 2015; Perez and Okonofua, 2022), leading to increased disciplinary actions and corresponding feedback from teachers. As this feedback accumulates over time, parents may gradually recognize and internalize this label, which in turn amplifies their skepticism and distrust toward teachers’ disciplinary practices, ultimately causing them to disengage from home-school collaboration.

## 6 The practical implications

To the best of our knowledge, this study is the first to empirically demonstrate the significant spillover effect of teacher discipline on parental trust judgments among rural Chinese parents. Specifically, the intensity of teachers’ disciplinary actions and the severity of students’ violations are associated with parents’ perceptions of teachers’ trustworthiness, which in turn predicts their trust in teachers. These findings have important theoretical and practical implications for establishing, maintaining, and enhancing home-school collaboration. First, this research provides novel evidence that teacher discipline significantly predicts parental trust, underscoring its critical role in shaping family-school relationships. Second, it reveals that discipline predicts parental trust by affecting their judgment of teachers’ trustworthiness, thereby reaffirming the core tenets of signaling theory within the context of educational management. Finally, the mediation effect of trustworthiness varies based on the severity of student violations, with minor infractions yielding a more pronounced effect compared to severe violations. This finding aligns with the principles of just deserts theory. More critically, this necessitates that educators consider students’ prior disciplinary records when administering disciplinary actions. Specifically, for students who sporadically infringe upon rules, educators should adhere to the principle of proportionality in implementing disciplinary actions, ensuring that the intensity of the punishment aligns appropriately with the offense while avoiding both excessive leniency and harshness. Only through such an approach can trust between educators and parents be effectively established and sustained. Conversely, for students with a history of repeated rule violations, parents frequently harbor hostility and skepticism toward disciplinary practices. Regardless of how disciplinary actions are applied, such actions may diminish parental trust. Hence, at this juncture, educators should prioritize addressing parental concerns and restoring the home-school relationship, such as by enhancing communication and collaboration between the two parties.

## 7 Limitations and future recommendations

As with other studies, this research has several limitations that warrant careful consideration. Firstly, the current study uses shortened scales with fewer items to evaluate the core variables, which might affect the reliability and validity of the variables and thereby weaken the internal and external validity of the results. Therefore, it is necessary for future research to adopt more complete and comprehensive psychological scale tools to assess variables and reverify the validity and robustness of the current research findings. Secondly, the current study employs a cross-sectional questionnaire design, which precludes the establishment of causal relationships between variables. Future research should therefore adopt more rigorous longitudinal or experimental designs to further investigate and establish causality between variables (Engeler and Raihani, 2024; Zhang et al., 2025c; Zhang and Qi, 2024). Thirdly, the participants in this study were exclusively from a county town in rural China, potentially compromising the representativeness of the sample and limiting the generalizability of the findings. Future studies should aim to validate these results using nationally representative data from publicly available databases, such as the China Education Panel Survey (CEPS) Database. Finally, parents’ trust in teachers is a multifaceted construct encompassing cognitive, attitudinal, and behavioral dimensions. The current study assessed parental trust using only two items, which may not fully capture the complexity of this concept. Future research should employ more comprehensive and diverse measures to evaluate this construct.

In addition, future research can be further expanded and deepened in the following areas. On the one hand, parental trust refers to a positive expectation formed by parents based on teachers’ care and concern for students. Teacher care plays a pivotal role in the establishment and maintenance of parental trust. Consequently, future studies should investigate both the extent to which and the mechanisms through which educational discipline grounded in the concept of care influences parental trust. On the other hand, the interpersonal trust that parents place in teachers constitutes a mutually supportive relationship, which necessitates communication, interaction, and mutual support, particularly in addressing minors’ disciplinary infractions. At this juncture, home-school communication emerges as a critical avenue for facilitating home-school co-education. Therefore, future research should delve into the function of home-school communication in mediating the relationship between educational discipline and parental trust.

## 8 Conclusion

This study, employing an online questionnaire, is the first to systematically investigate both the presence and mechanisms by which teacher discipline predicts parental trust among rural parents in China. The findings reveal that (1) the intensity of teacher discipline significantly and negatively predicts parents’ interpersonal trust in disciplinary teachers; (2) the intensity of discipline indirectly predicts parental trust through all sub-dimensions of trustworthiness—namely ability, benevolence, and integrity; (3) the mediating effect of these sub-dimensions of trustworthiness is more pronounced in low-breach scenarios compared to high-breach scenarios.

## Data Availability

The raw data supporting the conclusions of this article will be made available by the authors, without undue reservation.
